# Covalent immobilization of metal–organic frameworks onto the surface of nylon—a new approach to the functionalization and coloration of textiles

**DOI:** 10.1038/srep22796

**Published:** 2016-03-07

**Authors:** Ming Yu, Wanxin Li, Ziqiang Wang, Bowu Zhang, Hongjuan Ma, Linfan Li, Jingye Li

**Affiliations:** 1 CAS Center for Innovation in Advanced Nuclear Energy, Shanghai Institute of Applied Physics, Chinese Academy of Sciences, Shanghai, 201800, P. R. China

## Abstract

The prevention of refractory organic pollution caused by conventional dyeing and the development of new fabrics with various functions are two issues to be solved urgently in the field of textile fabrication. Here, we report a new environmentally friendly route for the simultaneous coloration and functionalization of textiles by the covalent immobilization of a metal–organic framework, Cr-based MIL-101(Cr), onto the surfaces of nylon fabrics by co-graft polymerization with 2-hydroxyethyl acrylate initiated by γ-ray irradiation. The Cr(III) clusters color the nylon fabric, and the color intensity varies with the MIL-101 content, providing a “green” textile coloration method that is different from conventional dyeing processes. An X-ray diffraction (XRD) analysis shows that the nanoporous structure of the original MIL-101 particles is retained during radiation-induced graft polymerization. Numerous nanopores are introduced onto the surface of the nylon fabric, which demonstrated better sustained-release-of-aroma performance versus pristine nylon fabric in tests. The modified fabrics exhibit laundering durability, with MIL-101 nanoparticles intact on the nylon surface after 30 h of dry cleaning.

Textiles, which are unique and necessary to human beings, are used in clothing as well as in decorative and industrial applications. Regardless of our long association with textiles and their manufacture on an industrial scale, many problems exist for scientists to solve. First, the textile industry has been one of the main sources of environmental pollution, especially through conventional dyeing techniques that use organic dyes and produce refractory organic pollutants^1^,^2^. Therefore, environmentally friendly coloration techniques are urgently needed for textiles. A further problem stems from the rapid increases in many people’s quality of life, which necessitates that textiles exhibit functionality in addition to their normal domestic and clothing properties. Sustained-release-of-aroma^3^,^4^, self-cleaning^5^,^6^, antimicrobial^7^, antistatic^8^, flame-retardant^9^,^10^, insect-repellent^11^, electromagnetic-shielding^12^, waterproof, and moisture-permeable^13^,^14^ properties are various functional textile characteristics that have recently received much attention.

Advancements in nanotechnology have provided new ways to functionalize textiles. Among the wide range of available nanomaterials are metal–organic frameworks (MOFs), which comprise an expanding class of porous crystalline materials built from nodes of metal ions connected by organic linkers^15^,^16^. These materials have shown promise in applications such as gas storage^17^,^18^, molecular separation^19^,^20^,^21^, heterogeneous catalysis^22^,^23^, drug delivery^24^, and chemical sensing^25^ because of their high surface areas, tunable pore sizes, and chemical versatility. Thus, the immobilization of MOFs onto textiles is a promising way to fabricate functional textiles, and one method involves the deposition of MOFs onto polymer solutions and the production of functional nonwoven fabrics by an electrospinning procedure^26^,^27^. Another method is the *in-situ* growth of MOFs on carboxylic textiles^28^,^29^. Besides the potential for textile functionalization, the immobilization of MOFs on textiles may provide a new environmentally compatible method for textile coloration because MOFs exhibit various colors according to their core metal clusters. Therefore, the immobilization of MOFs onto textiles has the potential to solve the two main problems of the textile industry.

Because of the nanoparticulate nature of MOFs, it can be difficult to firmly immobilize them on a textile. The *in-situ* growth method can only be applied to textiles containing carboxyl groups, and the choice of substrates is limited. Methods such as coating and deposition can only establish weak physical connections between the MOFs and the substrates, and the functionality of the composite materials is not typically durable with use. Radiation-induced graft polymerization (RIGP) can introduce functional groups onto textiles via strong covalent bonds, which can then be used to prepare functional durable textiles^30^,^31^,^32^. Compared to organic monomers, it is much more difficult to immobilize MOF particles onto textiles directly with covalent bonds because of the steric hindrance. A long, flexible, chain-like linker is needed as a bridge between the MOF particles and the textile, just as thread is needed to sew buttons onto clothes.

Herein, we report a novel approach for covalently immobilizing MOF particles onto textiles. MIL-101 (Cr) (denoted as MIL-101), a typical MOF which comprises a nontoxic chromium(III)^33^ cluster and benzene-1,4-dicarboxylate ligand and possesses a high surface area and good chemical stability^34^,^35^,^36^,^37^, was coated onto nylon fabric with 2-hydroxyethyl acrylate (HEA); then, co-graft polymerization was initiated by γ-ray irradiation, as illustrated in Fig. 1. Owing to the abundance of benzene rings in MIL-101, free radicals are generated on its surface upon γ-ray irradiation, which also occurs on the nylon fabric. The subsequent graft polymerization of HEA (PHEA) establishes a network of covalent bonds that link the MIL-101 nanoparticles and the nylon.

## Results

### Radiation Effect of the MOF

The covalent bonding of PHEA graft chains to textiles has been previously demonstrated^31^. The key to the link between MIL-101 and the nylon fabric realized by the PHEA graft chain is the generation of free radicals in MIL-101 under γ-ray irradiation, which initiates the graft polymerization of HEA on MIL-101.

Electron-spin resonance (ESR) was used to study the species generated and the decay kinetics of the free radicals of MIL-101 (Supplementary Fig. S1). From the ESR curves and the yield of free radicals, the species generated by the irradiation of MIL-101 are benzoyl radicals^38^. The concentration of free radicals decays with time upon storage, which demonstrates the reactivity of the benzoyl radicals. The mechanism of the generation of MIL-101 free radicals under γ-ray irradiation is shown in Supplementary Fig. S2.

To further study the graft polymerization of HEA on MIL-101, we combined them under γ-ray irradiation (experimental details, Supplementary Information). Figure S3 shows the mechanism of the graft polymerization of HEA on MIL-101. Figure S4a shows the effect of the HEA concentration on the degree of grafting (DG). The DG increases with the HEA concentration, which is similar to conventional graft polymerization. The Fourier-transform infrared (FT-IR) spectra of the pristine MIL-101 and MIL-101-g-PHEA are shown in Fig. S4b. In the grafted MIL-101 spectrum, a new band at 1720 cm^−1^ due to the stretching vibration of the carboxyl group^39^ confirms the success of the grafting. The thermogravimetric (TG) analysis curves of the pristine and MIL-101-g-PHEA are shown in Fig. S5. The residue weight of the grafted MIL-101 is lower than that of the pristine nylon because the PHEA graft chains degrade more thoroughly. The scanning electronic microscopy (SEM) images of the pristine MIL-101 and MIL-101-g-PHEA are shown in Fig. S6. The surface of the pristine MIL-101 is smooth, whereas the surface of the grafted MIL-101 is rough because of the accumulation of grafted PHEA chains.

### Co-graft Polymerization of the MOF

**Figure 2:**
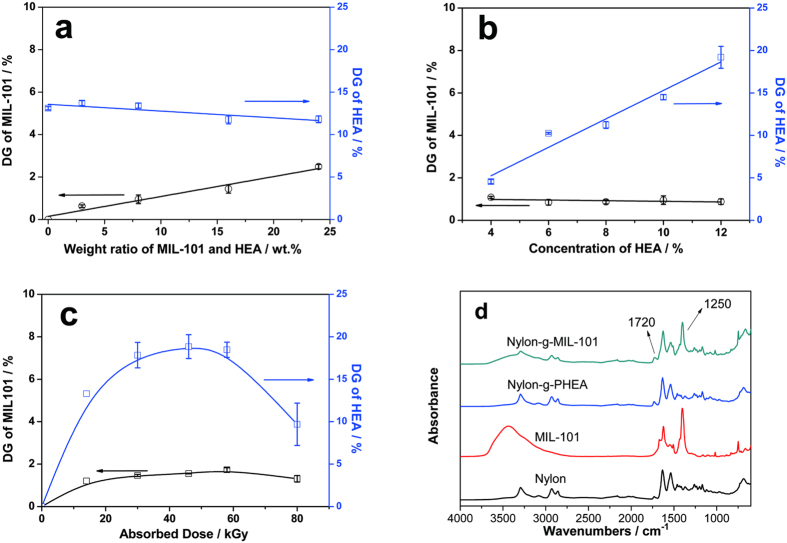
The kinetics of RIGP of MIL-101 and HEA onto nylon. **(a)** The effect of the weight ratio of MIL-101 to HEA on the DG (absorbed dose = 30 kGy; dose rate = 1.8 kGy/h; initial HEA concentration = 10% (v/v)). **(b)** The effect of the HEA concentration on the DG (absorbed dose = 30 kGy; dose rate = 1.8 kGy/h; MIL-101/HEA = 8:1 (w/w%)). **(c)** The effect of the absorbed dose on the DG (dose rate = 1.8 kGy/h; HEA concentration = 10% (v/v); MIL-101/HEA = 8:1 (w/w%)). (**d)** FT-IR spectra of pristine nylon fabric, MIL-101, nylon-g-PHEA (DG_HEA_ = 13.0%), and nylon-g-MIL-101 (DG_HEA_ = 11.4% and DG_MIL-101_ = 2.5%).

The two-unit co-graft polymerization of MIL-101 and HEA on nylon fabric establishes a network between the MIL-101 particles and the nylon. The kinetics of co-graft polymerization were studied. Figure 2a shows the effect of the weight ratio of MIL-101 to HEA on the DG. Interestingly, the DG of MIL-101 linearly increases with the increase in the weight ratio of MIL-101, whereas the DG of HEA slightly decreases. This is because the free radicals generated on MIL-101 couple with the free radicals on the growing PHEA graft chains on the nylon fabric, which terminates the propagation of the graft chain. In this manner, the covalent linking of MIL-101 and the nylon fabric via bridging by the PHEA graft chains is accomplished. Because the particle sizes of MIL-101 are in the nanometer-to-micrometer range, there should be many free radicals on the surface of a single MIL-101 particle, and MIL-101 reacts as a giant multifunctional crosslinking agent. Therefore, co-graft polymerization generates a three-dimensional network that firmly immobilizes the MIL-101 particles. This kinetic regularity is in accordance with our proposed mechanism for the immobilization of MIL-101. Figure 2b reveals the relationship between the DG and the HEA monomer concentration. The DG of MIL-101 changes very slightly with increasing monomer concentration. On the other hand, the DG of HEA linearly increases with the increase in the monomer concentration. This is because the increase in the monomer concentration enables more monomer molecules to be converted to grafted chains during graft polymerization. In the curves shown in Fig. 2c, the DGs of MIL-101 and HEA both increase with increasing absorbed dose because there are more generated free radicals to initiate graft polymerization. However, both DGs begin to decline when the absorbed dose reaches a certain critical value (approximately 50 kGy). It is considered that the absorption of a high dosage leads to a degradation in the graft chains. Figure 2d presents the FT-IR spectra of the pristine nylon fabric, the as-prepared MIL-101, the nylon fabric grafted with HEA only (denoted as nylon-g-PHEA, DG_HEA_ = 13.0%), and the nylon fabric grafted with both HEA and MIL-101 (denoted as nylon-g-MIL-101, DG_HEA_ = 11.4% and DG_MIL-101_ = 2.5%). The existence of the PHEA graft chains on nylon-g-MIL-101 is confirmed by the absorption bands at 1720 cm^−1^ in the spectra of the grafted fibers, which correspond to the stretching vibrations of the carboxyl groups^36^. The successful immobilization of MIL-101 is confirmed by the new band in the spectrum of nylon-g-MIL-101 at 1250 cm^−1^, ascribed to the benzoic-acid structures in MIL-101. Because the chemical structure of MIL-101 is typical among MOF materials, the immobilization method applied to MIL-101 in this study is easy to popularize and apply to other MOF materials.

### Morphology and Coloring of the Functionalized Textiles

**Figure 3:**
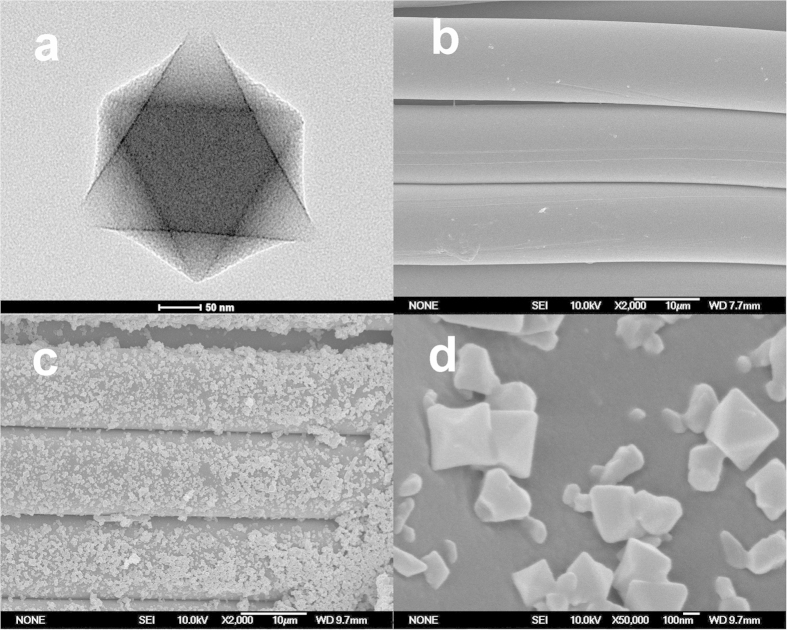
Micro-morphology of MIL-101 and nylon-g-MIL-101. TEM image of as-prepared MIL-101 **(a)**. SEM image of pristine nylon fabric **(b)**. SEM images of nylon-g-MIL-101 (**c:** ×2000; **d:** ×50000).

A transmission electron microscopy (TEM) image of the as-prepared MIL-101 is presented in Fig. 3a. MIL-101 exhibits an octahedral crystal structure, as previously described^31^. SEM images of pristine nylon and nylon-g-MIL-101 are presented in Fig. 3b–d. The surface of the pristine nylon fiber is smooth, whereas MIL-101 particles immobilized on the nylon fiber can be seen on the surface of nylon-g-MIL-101. Compared to the smooth surfaces of the as-prepared MIL-101 particles, the surfaces of the MIL-101 particles immobilized on nylon fabric are rougher because of the presence of the PHEA graft chain. The SEM images confirm that the MIL-101 particles are immobilized on the nylon fabric.

**Figure 4:**
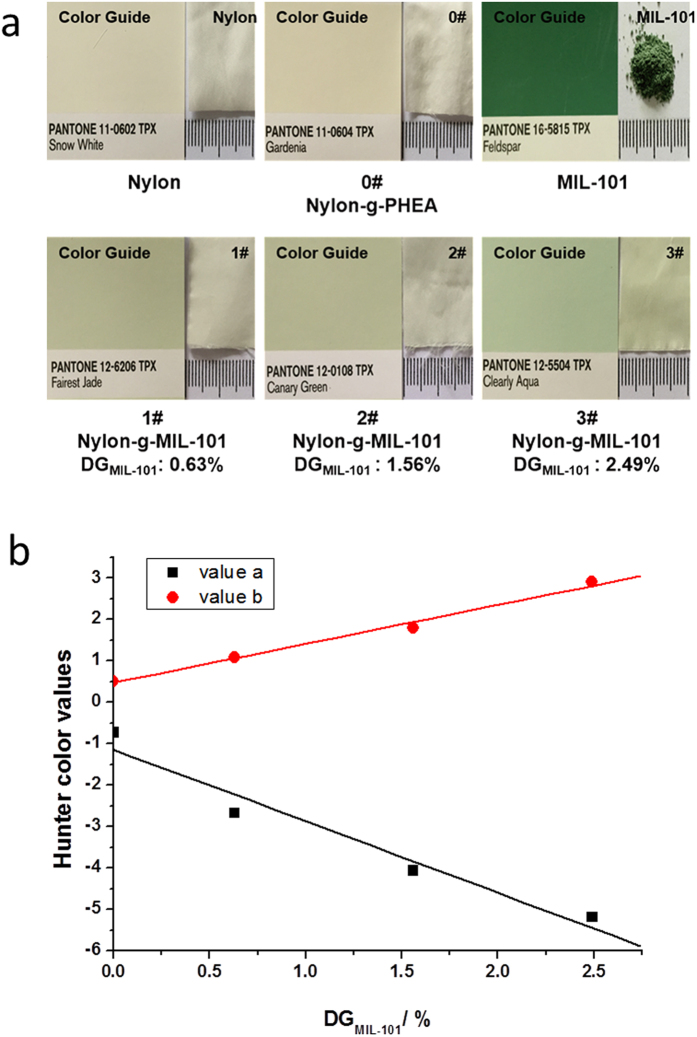
The measurements of the color of pristine and modified nylon fabrics. (**a)** Standard Colorimetric Test results for nylon fabric, MIL-101, nylon-g-PHEA (DG_HEA_ = 13.0%), and nylon-g-MIL-101 with different DGs (DG_MIL-101_ = 0.6%, 1.6%, and 2.5%). **(b)** The effect of DG_MIL-101_ on Hunter Lab values of the textiles.

The results of standard colorimetric tests using the Pantone cards of the pristine nylon fabric, MIL-101, nylon-g-PHEA (DG_HEA_ = 13.0%), and nylon-g-MIL-101 with different DGs (DG_MIL-101_ = 0.6%, 1.6%, and 2.5%) are presented in Fig. 4. The images show the snow-white color of the pristine nylon. After grafting with HEA, the color changes to light yellow (i.e., gardenia in the color guide). The color of MIL-101 is dark green (feldspar in the color guide), and the color of the nylon-g-MIL-101 is green owing to MIL-101 immobilization and darkens with the increasing DG of MIL-101. To study the effect of the immobilization of MIL-101 on the color of the nylon fabric objectively, the Hunter Lab values of the specimens were measured. Table S1 (Supplementary Information) shows the Hunter Lab values of the nylon fabric, nylon-g-PHEA (DG_HEA_ = 13.0%), and nylon-g-MIL-101 with different DGs (DG_MIL-101_ = 0.6%, 1.6%, and 2.5%). An increase in the L value means that the luminosity of the nylon fabrics increases with increasing DG_MIL-101_. The decrease in the a value suggests an increase in the green color, and an increase in the b value suggests an increase in the yellow color. It can be found that the a value of the nylon fabrics decreases with increasing DG_MIL-101_, and the b value increases with increasing DG_MIL-101_. This is consistent with the results of the standard colorimetric tests using Pantone cards. Figure 4b shows the linear relationship between changes in the a and b values and DG_MIL-101_, indicating that the color of nylon-g-MIL-101 can be controlled by changing the DG of MIL-101. Since MOFs have different colors depending on the central metal ions, textiles can be colored by the immobilization of different MOFs, and the intensity can be controlled by changing the DGs. This provides a new method for the coloration of textiles that is quite different from conventional dyeing using organic dyes.

The advantages of the RIGP method are that no chemical initiator is needed^40^ and the products are pure, which can greatly reduce the use of chemical reagents in postprocessing and is beneficial for environmental protection. Moreover, RIGP can be carried out at room temperature. Compared with the conventional grafting method, RIGP can save energy and is conducive to the environment. Ethanol is the solvent used in this system. The ones needing postprocessing are mainly unreacted MOF particles and the PHEA homopolymer. The unreacted MOF particles can be easily recovered by precipitation, centrifugation, and other methods. Further, PHEA is a nontoxic and biodegradable polymer^41^. Therefore, compared with the conventional organic dyeing method for refractory organic matter, which will cause serious damage to the environment^42^, the immobilization procedure is much more environmentally friendly and can be a “green” alternative.

### Nanoporosity and Sustained Release of Aroma

**Figure 5:**
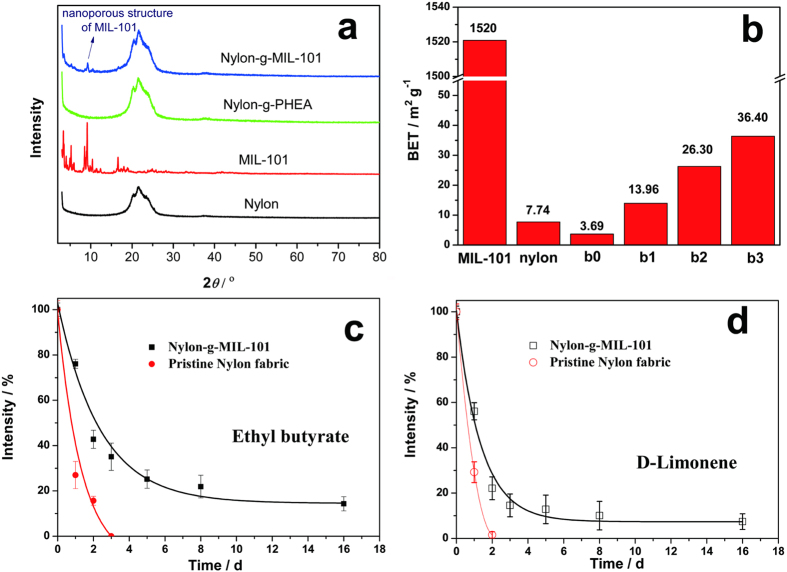
Nanoporosity and aroma sustained-release properties of nylon-g-MIL-101. (**a)** XRD curves of pristine nylon fabric, MIL-101, nylon-g-PHEA (DG_HEA_ = 13.0%), and nylon-g-MIL-101 with different DGs (DG_MIL-101_ = 0.6%, 1.6%, and 2.5%). **(b)** BET analysis results of as-prepared MIL-101, nylon fabric, nylon-g-PHEA (b0), and nylon-g-MIL-101 with different DGs ((b1) DG_MIL-101_ = 0.6%; (b2) DG_MIL-101_ = 1.6%; and (b3) DG_MIL-101_ = 2.5%). **(c)** Sustained release of ethyl butyrate on the pristine nylon fabric and nylon-g-MIL-101 (DG_MIL-101_ = 2.5%). **(d)** Sustained release of D-limonene on the pristine nylon fabric and nylon-g-MIL-101 (DG_MIL-101_ = 2.5%).

X-ray diffraction (XRD) was used to investigate the crystal structures of the pristine nylon fabric, as-prepared MIL-101, nylon-g-PHEA, and nylon-g-MIL-101 (Fig. 5a). The peak at 10° is characteristic for MIL-101 and is attributed to its nanoporous structure^31^. After graft polymerization, the peak at 10° appears in the pattern of nylon-g-MIL-101. This verifies the successful immobilization of MIL-101 on the nylon fabric and the preservation of the nanoporous structure during RIGP when abundant nanopores should have been introduced onto the nylon fabric. To confirm this, Brunauer–Emmett–Teller (BET) surface-area measurements were performed (Fig. 5b). The surface area of the nylon substrate significantly increases with the immobilization of MIL-101 particles. The specific surface area also increases with the DG of MIL-101. The theoretical relationship between the surface area of nylon-g-MIL-101 and the DG of MIL-101 was calculated according to the surface area of the as-prepared MIL-101, the surface area of the pristine nylon, and the DG of MIL-101 (Supplementary Fig. S7). The actual values were nearly the same as the theoretical values, which confirms that the surface area of MIL-101 slightly changes during RIGP.

Hydrophilic D-limonene (DL) and hydrophobic ethyl butyrate (EB) were used as model compounds to investigate the sustained-release-of-aroma properties of the modified nylon fabrics. The results are shown in Fig. 5c,d. DL and EB were directly applied onto the surface of the fabrics. After two or three days, all of the aromatics on the pristine nylon fabric evaporated, whereas a substantial quantity of the aromatics remained on nylon-g-MIL-101 after 16 days. Obviously, the improvement in the sustained release of aromas by the modified nylon fabric is due to the abundance of nanopores introduced during RIGP. The volatilization coefficient of DL on nylon-g-MIL-101 was −0.42, which is almost half of the volatilization coefficient on the pristine nylon fabric (−0.74). Further, the volatilization coefficients of EB on the nylon-g-MIL-101 and pristine nylon samples were similar. These improved properties are in accordance with the changes in the surface area of the nylon fabric presented in Fig. 5b.

### Laundering Durability of the Modified Fabrics

**Figure 6:**
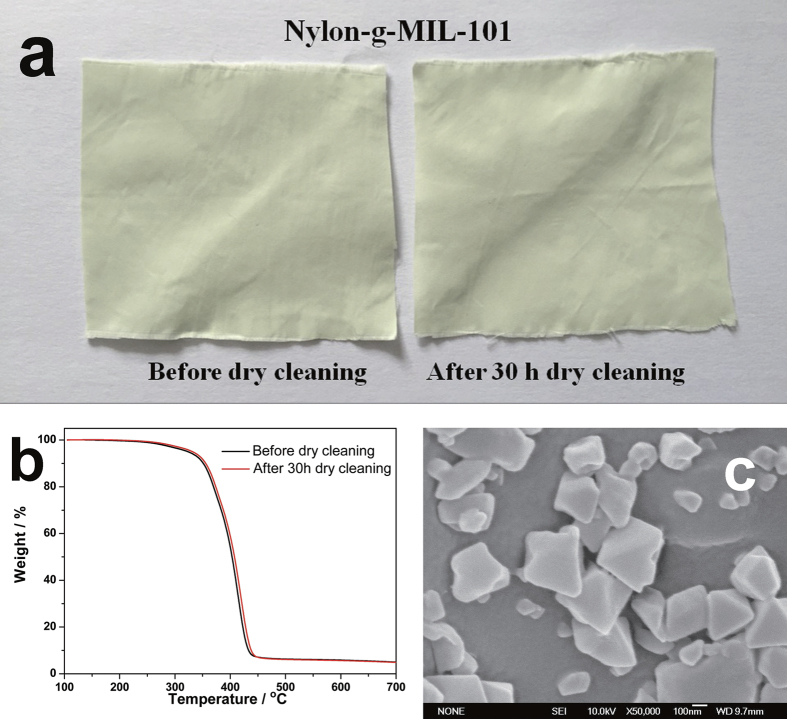
Dry-cleaning durability of nylon-g-MIL-101 (DG_MIL-101_ = 2.5%). Images of nylon-g-MIL-101 samples after 30 h dry cleaning and without dry cleaning (**a**). TG curves of nylon-g-MIL-101 samples after30 h dry cleaning and without dry cleaning (**b**). SEM image of nylon-g-MIL-101 after 30 h dry cleaning (**c**).

To investigate the robustness of the immobilization of MIL-101 particles, dry-cleaning tests were performed using tetrachloroethylene as the organic solvent. Images of nylon-g-MIL-101 before and after 30 h of dry cleaning are shown in Fig. 6a. After a 30 h treatment, the color of the sample was nearly unchanged. The TG curves (Fig. 6b) of the two samples nearly coincide. In addition, the Hunter Lab values of the fabric after the dry-cleaning test are almost the same as the values measured before dry cleaning (Table S2, Supplementary Information). From the SEM image (Fig. 6c) of nylon-g-MIL-101 after 30 h of dry cleaning, most of the MIL-101 particles still adhered to the nylon fabric. All of these results highlight the strength of the covalent bonds between the MIL-101 particles and the nylon fabric bridged by the PHEA graft chains and demonstrate that the MIL-101 particles survive on the nylon fabrics after 30 h of dry cleaning.

## Discussion

In conclusion, MIL-101 particles were firmly immobilized onto nylon fabric by covalent bonds via RIGP with HEA. This method provides a new approach for the coloration of textiles. The nanoporous structure of MIL-101 was maintained during RIGP, and an abundance of nanopores were introduced onto the nylon fabric, which significantly improves the sustained-release-of-aroma properties of the fabric. The covalent bonds were very strong; therefore, most of the immobilized MIL-101 particles adhered to the nylon fabric, even after 30 h of dry cleaning, suggesting that the functionalized nylon fabrics have good durability.

## Methods

### Materials

The synthesis and evaluation of MIL-101 particles is according to the literature^37^. MIL-101 was synthesized by hydrothermal reaction of benzene-1,4-dicarboxylic acid (164 mg, 1 mmol), Cr(NO_3_)_3_·9H_2_O (400 mg, 1 mmol), hydrofluorhydric acid (5 M, 0.2 mL, 1 mmol) in H_2_O (4.8 mL, 265 mmol) at 220 °C for 8 h. The XRD results (Fig. 5a) proved the MIL-101 had been successfully synthesized. The evalution 2-Hydroxyethyl acrylate (HEA) with a purity of 96% and D-limonene with a purity of 95% were purchased from Tokyo Chemical Industry Co., Ltd. Analytical grades of ethanol, tetrachloroethylene, acetone and ethyl butyrate were purchased from Sinopharm Chemical Reagent Co., Ltd. All these materials were used without further purification.

### Radiation-induced co-graft polymerization of HEA and MIL-101 particles onto nylon fabric

Monomer solutions were prepared by dissolving HEA in ethanol. MIL-101 nanoparticles were weighed and added into the monomer solution and sheared (10000 rpm) to prepare the dispersion. The dispersion was coated onto the nylon fabric, which was then put into a sealed tube and bubbled with nitrogen gas for 15 min to remove oxygen. The samples were irradiated by a ^60^Co source at room temperature for different doses. The irradiated samples were extracted with acetone for 72 h in a Soxhlet apparatus to remove the homopolymer. Then, the functionalized nylon fabrics were vacuum dried prior to further measurements.

### Determination of DG

Because the central metal ion of MIL-101 is Cr, the DG of MIL-101 on nylon can be calculated according to the Cr content of the nylon-*g*-MIL-101, which can be determined after microwave digestion of the co-graft polymer and measurement via inductively coupled plasma–atomic emission spectrometry (ICP-AES). ICP-AES measurements were performed on an Optima 8000 instrument (PerkinElmer, U.S.). The DG of MIL-101 on the nylon fabric was calculated using Eq. (1):


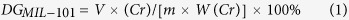


where *V* is the volume of the solution for the microwave digestion of MIL-101; *m* is the weight of the MIL-101 sample; (Cr) is the concentration of Cr in the microwave digestion solution; and *W*(Cr) is the mass fraction of Cr in MIL-101.

The total DG can be calculated by the change of the weight of the nylon fabric before and after grafting. The DG of HEA on nylon fabric was calculated using Eq. (2):


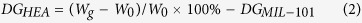


where *W*_*0*_ and *W*_*g*_ are the weights of the nylon fabric before and after grafting.

### Measurements of the chemical structure and physical properties of the materials

FT-IR spectra were obtained on a BRUKER TENSOR 27 FT-IR spectrometer. The MIL-101 particles were pressed into a pellet with KBr. The pristine and functionalized nylon fabrics were measured using the attenuated total reflection (ATR) method. XRD analysis was performed on a BRUKER D8 Advance XRD instrument equipped with Cu Kα radiation (λ = 1.54 Å). TG analysis was performed on a TG 209 F3 Tarsus (NETZSCH, Germany) instrument from 100 to 750 °C at a heating rate of 10 °C·min^−1^. SEM analysis was performed on a JEOL JSM-6700F SEM instrument. The BET surface area analysis was performed on a V-sorb 2800TP surface area and porosimetry analyzer.

### Measurements of the color of the textiles

The Hunter Lab values of the textiles were measured with Color measurement instrument (X-rite, COLOR-EYE7000A). Colorimetric analysis was also carried out by using Pantone colorimetric cards.

### Fragrance sustained-release property test

The fragrance sustained-release test was performed on a Hitachi U-3900 UV-Vis instrument. First, the perfumes (EB and DL) were added dropwise to the regularly shaped and sized graft samples (about 4 × 4 cm^2^), respectively. The samples were placed in the ambient environment for 0, 1, 2, 3, 5, and 16 days. At the designated time, each sample was extracted in ethanol for 2 h and the content of the fragrance retained in the fabric was measured by UV-Vis absorbance.

### Dry-cleaning durability test

The dry-cleaning durability evaluation was carried out according to the American Association of Textile Chemists and Colorists (AATCC) Test method 132-2004. The temperature of the tests is 30 °C. Tetrachloroethylene was used as the organic solvent.

## Additional Information

**How to cite this article**: Yu, M. *et al.* Covalent immobilization of metal-organic frameworks onto the surface of nylon-a new approach to the functionalization and coloration of textiles. *Sci. Rep.* **6**, 22796; doi: 10.1038/srep22796 (2016).

## Supplementary Material

Supplementary Information

## Figures and Tables

**Figure 1:**
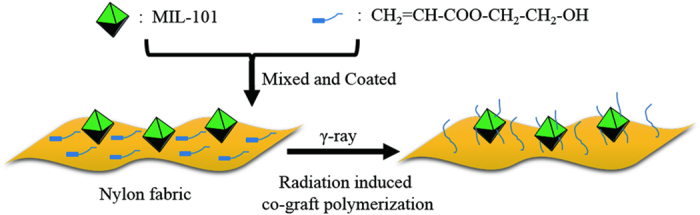
A schematic of the fabrication process. Mechanism of the preparation of nylon fabrics immobilized with MOFs by radiation-induced graft polymerization.
