# Supplementary material for: Covalent immobilization of metal–organic frameworks onto the surface of nylon—a new approach to the functionalization and coloration of textiles
Source: Sci Rep. 2016 Mar 7;6:22796. doi: 10.1038/srep22796 (PMC4780033; doi:10.1038/srep22796)
Supplement: Supplementary Information [file srep22796-s1.pdf]

Supporting Information

Covalent Immobilization of Metal Organic Frameworks onto the Surface of Nylon—A New Approach to the Functionalization and Coloration of Textiles

Ming Yu, Wanxin Li, Ziqiang Wang, Bowu Zhang, Hongjuan Ma, Linfan Li and Jingye Li*

CAS Center for Excellence on TMSR Energy System, Shanghai Institute of Applied Physics, Chinese Academy of Sciences, Shanghai, 201800, P. R. China.

E-mail: jingyeli@sinap.ac.cn; Tel: +86 39194505

Dr. M. Yu and Mr. W.X. Li contribute equally to this work.

Methods

Study of the free radicals of MIL-101 initiated by irradiation. MIL-101 particles were irradiated by a ^{60}Co source for an absorbed dose of 30 kGy. The irradiated MIL-101 was stored under air atmosphere at room temperature for various lengths of time (0–30 days) and assessed using a FA 200 ESR instrument (JEOL Co.).

Reaction between HEA and MIL-101 particles initiated by irradiation. HEA was dissolved in ethanol in a tube and the MIL-101 particles were immersed in the solution. Then, the solution was bubbled with N_2 to remove oxygen and the tube was sealed and irradiated by a ^{60}Co source. The grafted MIL-101 particles were extracted with acetone for 72 h in a Soxhlet apparatus to remove the homopolymer. The samples were then vacuum dried before further measurements.

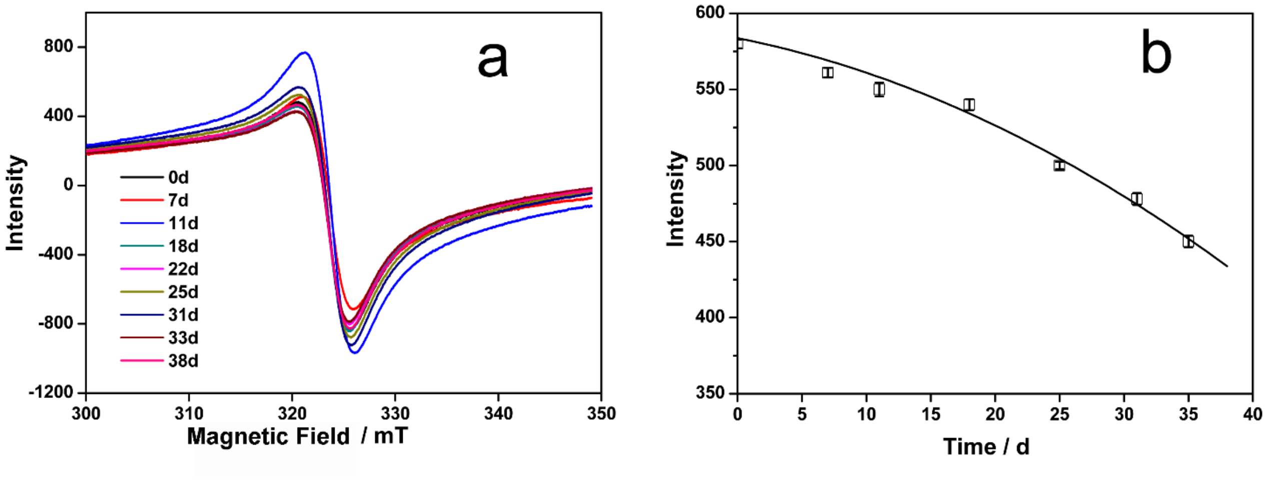

Figure S1│ ESR measuments of MIL-101 after irradiation. (a) ESR spectra of MIL-101 particles under radiation (scale of Y axis: relative intensity of ESR signals). **(b)** Decay of the free radicals in the MIL-101 generated by irradiation (30 kGy, 17 h air) (scale of Y axis: relative intensity of ESR signals).

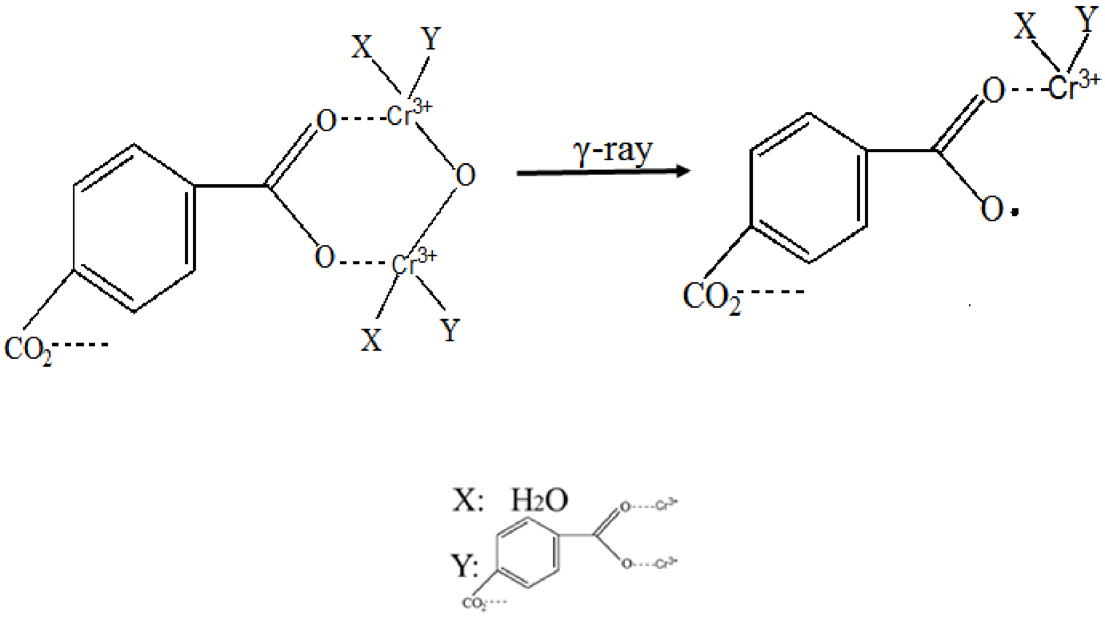

Figure S2│ The mechanism of the generation of the free radicals of MIL-101 under irradiation.

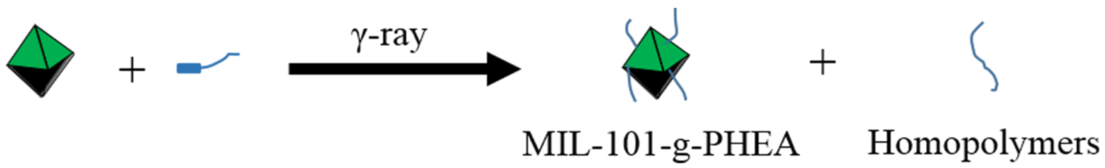

Figure S3 | The mechanism of irradiation-induced grafting of HEA on MIL-101 particles.

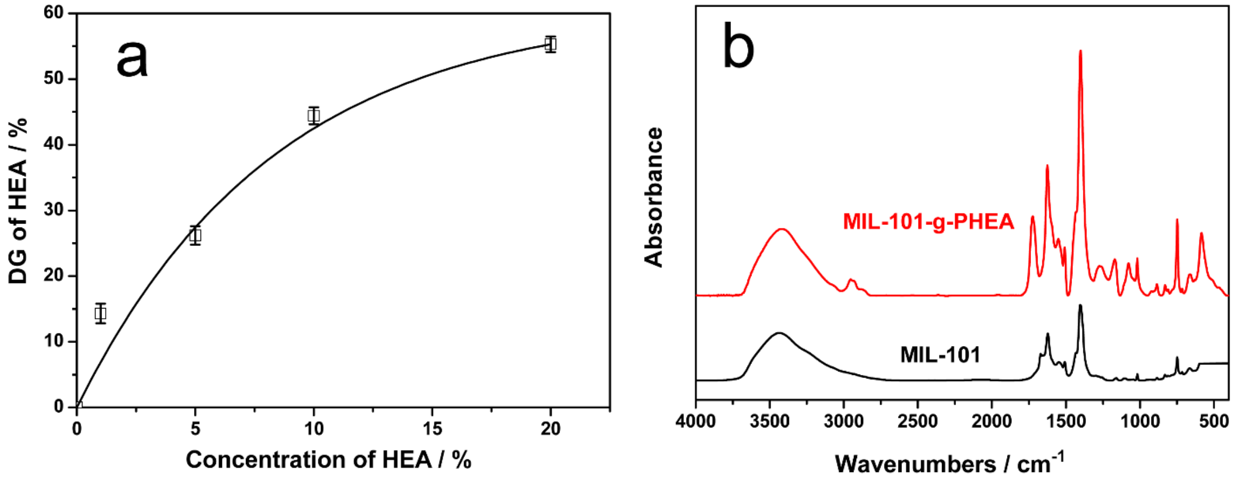

Figure S4 | The kinetics of the RIGP of HEA onto MIL-101. (a) The effect of the HEA concentration on the DG of the HEA grafted on the MIL-101. **(b)** FT-IR spectra of as-prepared MIL-101 and MIL-101 grafted with HEA.

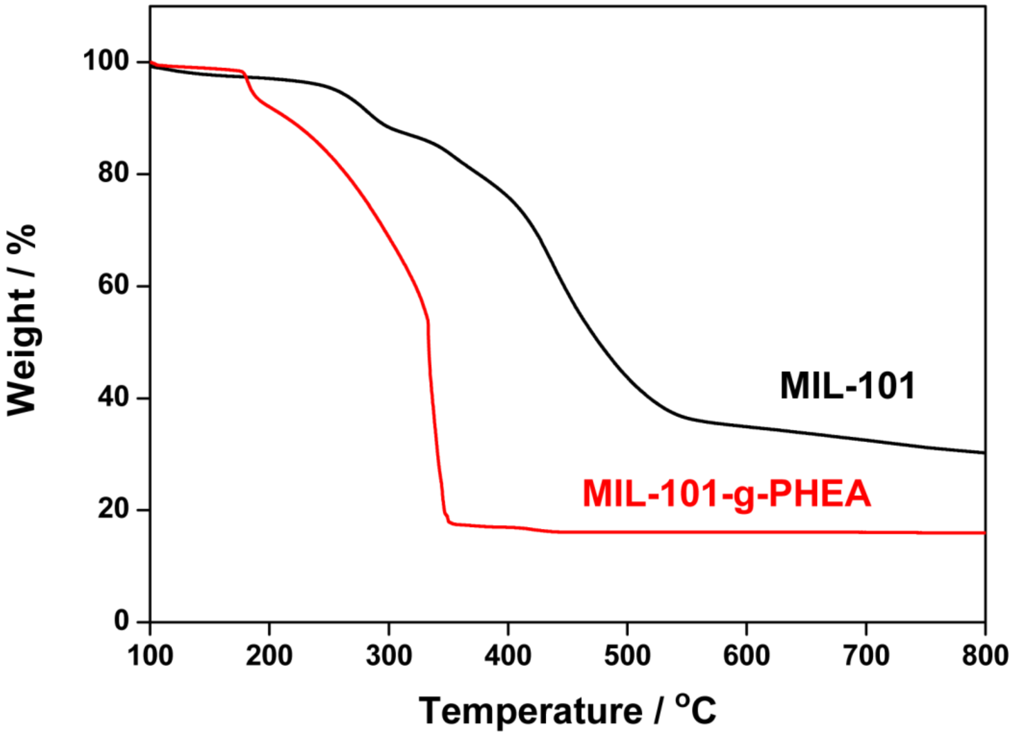

Figure S5│Thermal stability of MIL-101 and MIL-101 grafted with HEA. TG curves of as-prepared MIL-101 **(a)** and MIL-101 grafted with HEA **(b)**.

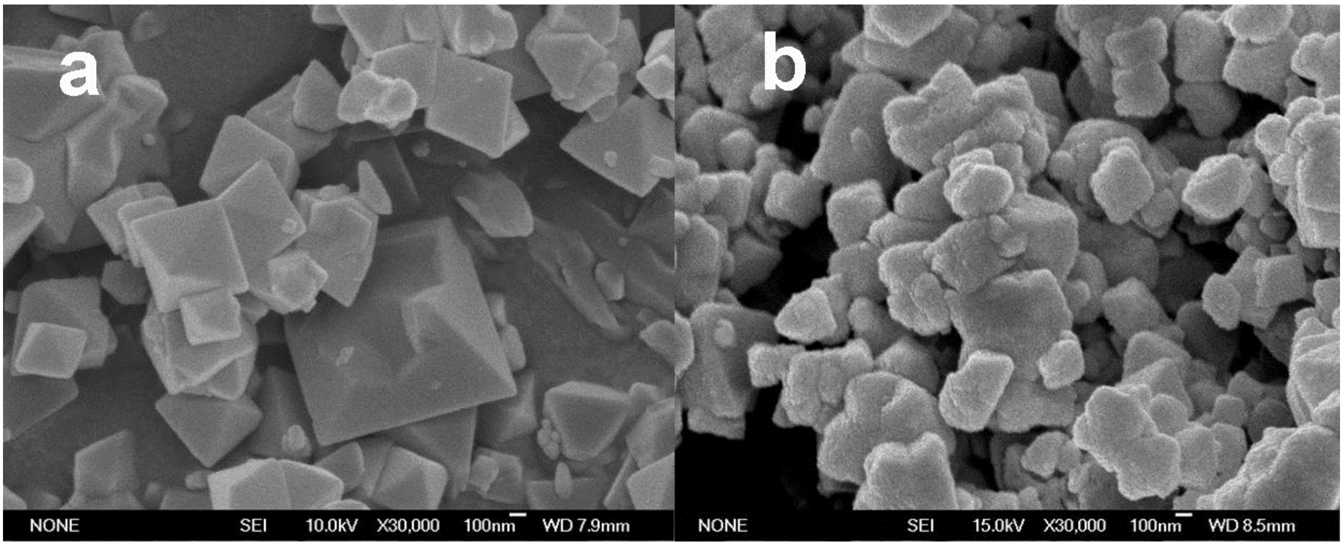

Figure S6│ SEM images of as-prepared MIL-101 (a) and MIL-101 grafted with HEA (b).

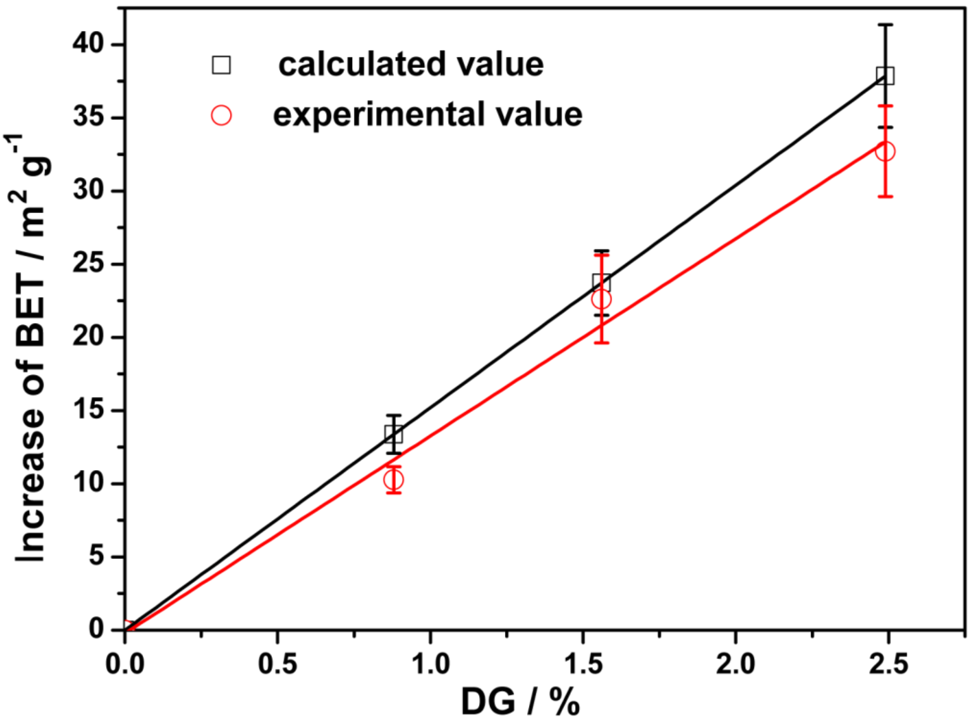

Figure S7│ Theoretical and actual values of the BET of nylon-*g*-MIL-101 as a function of the DG of MIL-101.

Table S1 │ The Hunter color values (L, a, b) of nylon fabric, nylon-g-PHEA (DG_{HEA} = 13.0%), and nylon-g-MIL-101 with different DGs ($DG_{MIL-101}$ = 0.6%, 1.6%, and 2.5%).

**Spicemens**	**Hunter color values**		
	**L**	**a**	**b**
nylon fabric	72.43	-0.79	0.35
nylon-g-PHEA (DG_{HEA} = 13.0%)	75.30	-0.72	0.51
nylon-g-MIL-101 ($DG_{MIL-101}$ = 0.6%)	76.34	-2.67	1.09
nylon-g-MIL-101 ($DG_{MIL-101}$ = 1.6%)	79.40	-4.07	1.79
nylon-g-MIL-101 ($DG_{MIL-101}$ = 2.5%)	79.98	-5.19	2.90

Table S2 │ The Hunter color values (L, a, b) of nylon-g-MIL-101 ($DG_{MIL-101}$ = 2.5%) before and after 30 h dry cleaning.

**Spicemens**	**Hunter color values**		
	**L**	**a**	**b**
Before dry cleaning	79.98	-5.19	2.90
After 30 h dry cleaning	79.55	-5.06	2.94
